# Early prediction of phenotypic severity in Citrullinemia Type 1

**DOI:** 10.1002/acn3.50886

**Published:** 2019-08-30

**Authors:** Matthias Zielonka, Stefan Kölker, Florian Gleich, Nicolas Stützenberger, Sandesh C. S. Nagamani, Andrea L. Gropman, Georg F. Hoffmann, Sven F. Garbade, Roland Posset, the Urea Cycle Disorders Consortium (UCDC) and the European Registry and Network for Intoxication type Metabolic Diseases (E‐IMD) Consortia Study Group

**Affiliations:** ^1^ Center for Pediatric and Adolescent Medicine Division of Pediatric Neurology and Metabolic Medicine University Hospital Heidelberg Im Neuenheimer Feld 430 69120 Heidelberg Germany; ^2^ Heidelberg Research Center for Molecular Medicine (HRCMM) Heidelberg Germany; ^3^ Department of Molecular and Human Genetics Baylor College of Medicine Texas Children's Hospital Houston Texas; ^4^ Children's National Health System The George Washington School of Medicine District of Columbia Washington

## Abstract

**Objective:**

Citrullinemia type 1 (CTLN1) is an inherited metabolic disease affecting the brain which is detectable by newborn screening. The clinical spectrum is highly variable including individuals with lethal hyperammonemic encephalopathy in the newborn period and individuals with a mild‐to‐moderate or asymptomatic disease course. Since the phenotypic severity has not been predictable early during the disease course so far, we aimed to design a reliable disease prediction model.

**Methods:**

We used a newly established mammalian biallelic expression system to determine residual enzymatic activity of argininosuccinate synthetase 1 (ASS1; OMIM #215700) in 71 individuals with CTLN1, representing 48 *ASS1* gene variants and 50 different, mostly compound heterozygous combinations in total. Residual enzymatic ASS1 activity was correlated to standardized biochemical and clinical endpoints available from the UCDC and E‐IMD databases.

**Results:**

Residual enzymatic ASS1 activity correlates with peak plasma ammonium and L‐citrulline concentrations at initial presentation. Individuals with 8% of residual enzymatic ASS1 activity or less had more frequent and more severe hyperammonemic events and lower cognitive function than those above 8%, highlighting that residual enzymatic ASS1 activity allows reliable severity prediction. Noteworthy, empiric clinical practice of affected individuals is in line with the predicted disease severity supporting the notion of a risk stratification‐based guidance of therapeutic decision‐making based on residual enzymatic ASS1 activity in the future.

**Interpretation:**

Residual enzymatic ASS1 activity reliably predicts the phenotypic severity in CTLN1. We propose a new severity‐adjusted classification system for individuals with CTLN1 based on the activity results of the newly established biallelic expression system.

## Introduction

Citrullinemia type 1 (CTLN1) is an autosomal recessive urea cycle disorder caused by deficiency of the cytosolic enzyme argininosuccinate synthetase 1 (ASS1‐D; MIM #215700) due to pathogenic variants in the *ASS1* gene located on chromosome 9q34.11. CTLN1 is one of the most common urea cycle disorders (UCDs) with an estimated overall incidence of 1 in 250,000 individuals.^1^, ^2^ The heterogeneous clinical presentation of individuals with CTLN1 includes severe and life‐threatening hyperammonemic events (HAEs) within the first 28 days of life (early onset, EO), a more variable phenotype presenting after the neonatal period (late onset, LO), and individuals with mild or no symptoms even in the absence of specific therapy. These are the major cornerstones of current clinical classification (EO, LO, asymptomatic) in individuals with CTLN1.^3^, ^4^ A meta‐analysis recently demonstrated that neonatal mortality in EO CTLN1 is still high and has not significantly improved for more than three decades, despite implementation of pharmacologic and extracorporeal detoxification for emergency treatment of HAEs, and low‐protein diet as well as nitrogen scavengers for maintenance treatment.^5^ Timely liver transplantation (LTx) might prove beneficial for severely affected individuals with regard to their cognitive outcome.^6^

Large observational natural history studies of UCDs in North America [Urea Cycle Disorders Consortium (UCDC; https://www.rarediseasesnetwork.org/cms/ucdc)] and Europe [European registry and network for intoxication type metabolic disease (E‐IMD; https://www.eimd-registry.org/)] have identified clinical variables, such as early disease onset, or biochemical variables, such as high initial peak plasma ammonium concentration (NH_4_^+^_max_), to be correlated with poor long‐term outcome.^7^, ^8^, ^9^, ^10^ However, phenotypic severity has not been predictable early during the disease course so far. This has hampered the development of severity‐adjusted therapeutic strategies to improve the health outcomes. The increasing identification of individuals with a potentially benign disease course by newborn screening programs for CTLN1 supports the need for a reliable prediction model to reduce the risk of over‐ and undertreatment. Based on a systematic data collection, currently more than 100 disease‐causing *ASS1* genetic variants are known; however, the impact of the genotype on the phenotypic presentation remains insufficiently understood.^11^ Given that residual enzymatic activities have already been reported to predict disease severity and survival rates in other inborn errors of metabolism, such as Farber disease and mucopolysaccharidosis type IIIA and VII,^12^, ^13^, ^14^ we hypothesized that ASS1 enzyme activity may correlate with disease severity in CTLN1.

Furthermore, since we recently demonstrated that diagnosis and therapy are important, but not the only factors that have an impact on the cognitive outcome in urea cycle disorders,^6^ we hypothesize that the underlying genotypic variability might have an important effect on the clinical outcome. Thus, we evaluated whether the residual enzymatic ASS1 activity, as determined by an innovative and newly established mammalian biallelic expression system, can predict the clinical disease course and neurological outcome. To this end, we systematically investigated all reported exonic variants from the worldwide largest cohort of individuals with CTLN1 reported in the UCDC and E‐IMD patient registries and correlated data with the individuals' biochemical, clinical, and neurological follow‐up data.

## Materials and Methods

### Eligibility criteria

Only individuals with CTLN1 confirmed by biallelic pathogenic variants in *ASS1* who were enrolled in the observational longitudinal studies UCDC (NCT00237315) and E‐IMD were included in this analysis. The data model of both registries, information on written informed consent as well as the follow‐up protocols used have been previously described in detail.^2^, ^6^ Requirements set forth by the ICMJE (International Committee of Medical Journal Editors) were met. All procedures followed were in accordance with the ethical standards of the Helsinki Declaration of 1975, as revised in 2013. Data were retrieved from the UCDC and E‐IMD electronic databases with the cut‐off date for data retrieval being October 10, 2018. The UCDC database is registered at the US National Library of Medicine (https://clinicaltrials.gov), whereas the E‐IMD registry is recorded on the German Clinical Trials Register (https://www.drks.de).

### Plasmids

To generate the tagged wildtype *ASS1* expression vectors, the ASS1 coding sequence was amplified by PCR with MYC‐ or FLAG‐tags introduced at the C‐ or N‐terminus using specific primer pairs and inserted into the BamHI and NotI restriction sites in the open‐reading frame of the eukaryotic expression vector pcDNA5 (Thermo Fisher Scientific). Pathogenic *ASS1* gene variants observed in individuals with CTLN1 within the E‐IMD and UCDC registries were introduced into the tagged *ASS1* expression vectors using the QuickChange II site‐directed mutagenesis kit (Agilent) according to the manufacturer's protocol. The wildtype *ASS1* expression vectors and the correct insertion of the mutations were confirmed by Sanger‐sequencing. The pSV‐*β*‐galactosidase control vector (Promega) was kindly provided by N. Himmelreich (Heidelberg University, Germany).

### Cell culture and transfections

COS‐7 cells were maintained as adherent cell cultures in 10 cm petri dishes in DMEM medium (Thermo Fisher Scientific) supplemented with 10% heat‐inactivated fetal bovine serum in a humified incubator at 37°C and 5% CO_2_. COS‐7 cells were transfected with 2.5 *µ*g of each FLAG‐ and MYC‐tagged ASS1‐plasmid and 1 *µ*g of *β*‐galactosidase reporter plasmid using Lipofectamin 2000 reagent (Thermo Fisher Scientific) according to the manufacturer's instructions. After 48 h, cells were washed two times with ice‐cold phosphate‐buffered saline (PBS), lysed and the lysates were utilized to perform qRT‐PCR, western blotting, and ASS1 enzyme assay.

### qRT‐PCR

RNA was extracted by standard procedure with TRIzol reagent (Invitrogen). Equal amounts from each sample (500–800 ng) were used for cDNA syntheses applying the Maxima first strand cDNA synthesis kit (Thermo Fisher Scientific). Real‐time qPCR was performed on a CFX Connect^™^ 180 Real‐Time cycler (Biorad) (denaturation step: 95°C for 25 sec, annealing and elongation step: 60°C for 30 sec) using SensiFast SYBR^™^ Hi‐ROX mix (Bioline) with the following primers:

#### Tagged‐ASS1‐plasmids (N‐terminus)

N‐FLAG_forward: 5′‐CTACAAAGACGATGACGACAAG‐3′

N‐MYC_forward: 5′‐GAAGAGGATCTGGGAGGTTCAGG‐3′

N‐tag_reverse: 3′‐CTTCTTCCTGGCTTCCTCG‐5′

#### Tagged‐ASS1‐plasmids (C‐terminus)

C‐tag_forward: 5′‐CCCACTGTCTCTCTACAATGAGG‐3′

C‐FLAG_reverse: 3′‐GTCGTCATCGTCTTTGTAGTCG‐5′

C‐MYC_reverse: 3′‐GTTTTTGTTCGCTGCCTCCTG‐5′

#### β‐Actin

Actin_forward: 5′‐CAACCTTCCTTCCTGGGCAT‐3′

Actin_reverse: 3′‐GATTTTCATCGTGCTGGGCG‐3′

The expression level of *β*‐actin was used for normalization.

### Western blot

Forty eight hours after transfection, COS‐7 cells were washed two times in ice‐cold PBS and lysed in 1× ice‐cold radioimmunoprecipitation buffer (600 mmol/L NaCl, 100 mmol/L TRIS‐HCl pH 7.4, 10 mmol/L EDTA, 2% Triton X‐100, 0.2% SDS, 1% sodium deoxycholate) followed by additional sonification. Hereafter, lysates were centrifuged at 13,000 x *g* and 4°C for 10 min, and supernatants were used for Western blotting according to standard laboratory protocols. For protein visualization, membranes were probed with the following primary antibodies: anti‐FLAG (1:2000, BioLegend), anti‐MYC (1:2000, Cell Signaling) and anti‐*β*‐Actin (1:2000, Sigma‐Aldrich).

### Spectrophotometric analysis of the ASS1 enzyme

Purified citrate synthase, malate dehydrogenase, and fumarase from porcine heart were purchased from Sigma‐Aldrich. Recombinant C‐terminally FLAG‐tagged argininosuccinate lyase protein was obtained from Creative Biomart. ASS1 enzyme activity was determined in transfected COS‐7 cell lysates (triplicates) in a buffer containing 10 mmol/L potassium phosphate, 10 mmol/L TRIS‐HCl, 2 mmol/L citrulline, 600 mU/mL of argininosuccinate lyase, 650 mU/mL fumarase, 660 mU/mL malate dehydrogenase, 400 mU/mL citrate synthase, 2 mmol/L aspartic acid, 330 *µ*mol/L NAD, 100 *µ*mol/L acetyl‐CoA and 2 mmol/L ATP, which was adjusted to pH 7.4 (25°C). ASS1 enzyme activity was determined as NAD reduction at *λ* = 340–400 nm. Lysates of COS‐7 transfected with empty pcDNA5 vector served as negative control. Negative control values were subtracted from ASS1 activities to adjust for ASS1 background activity.

To control for transfection efficacy, the ASS1 activities were normalized to the *β*‐galactosidase activity in each sample as determined by the *β*‐galactosidase enzyme assay system (Promega) according to the manufacturer's instructions. The adjusted ASS1 activities were normalized to the protein content in the respective samples. Residual activities are depicted as percentage of total (%) by dividing the normalized ASS1 activity of a specific mutational combination (homozygous/compound heterozygous) by the normalized wildtype ASS1 activities.

### Clinical variables used for data analyses

Data on the following numerical clinical variables were collected: NH_4_^+^_max_, peak plasma L‐citrulline concentration, number of HAEs (with NH_4_^+^_max_ > 100 *µ*mol/L) per year of observation (defined as time between date of birth and last regular visit), NH_4_^+^_max_ during the most severe HAE, and cognitive SDS at the most recent visit being calculated using the normative data from the standardization sample of each cognitive test. The most recent visit was chosen since this is the latest developmental data on patients which most likely represents the true outcome. For each Hamburg‐Wechsler‐Intelligenztest für Erwachsene (*n* = 1/34), Wechsler Adult Intelligence Scale (*n* = 1/34), Wechsler Abbreviated Scale of Intelligence (*n* = 7/34), Wechsler Intelligence Scale for Children (*n* = 3/34), and Wechsler Preschool and Primary Scale of Intelligence (*n* = 4/34) full scale IQ was selected. For Bayley Scales of Infant Development (*n* = 15/34) mental developmental index and cognitive scale were used. For Adaptive Behavior Assessment System (*n* = 3/34) the general adaptive composite was used.^15^

Data on following categorical clinical variables was also used for this study: disease onset (EO, LO, asymptomatic), presence or absence of movement disorder (dystonia and/or chorea and/or ataxia), tone change (muscular hypotonia and/or muscular hypertonia and/or spasticity), special education (self‐contained class as well as any supportive services), hepatocellular injury (alanine aminotransferase ≥ 250 U/L or aspartate aminotransferase ≥ 250 U/L), liver transplantation (LTx), and kidney dysfunction [full age spectrum glomerular filtration rate (FAS‐GFR) ≥ 90 mL/min/1.73 m^2^ vs. FAS‐GFR < 90 mL/min/1.73 m^2^].^16^, ^17^

For symptomatic individuals with reported HAE during the initial presentation/decompensation, biochemical data (NH_4_^+^_max_ and peak plasma L‐citrulline concentration) represent the highest value prior to initiation of treatment. For symptomatic CTLN1‐individuals without reported HAE during initial presentation, NH_4_^+^_max_ was defined as upper limit of normal range (50 *µ*mol/L). For asymptomatic individuals, NH_4_^+^_max_ and peak plasma L‐citrulline concentrations represent highest reported follow‐up values during the observation period as conservative approach; only untreated asymptomatic CTLN1‐inviduals were considered for analysis. We investigated the impact of the cumulative residual ASS1 enzymatic activity as determined by the established biallelic expression system on clinical outcome parameters outlined above. Mortality could not be examined due to a low number of deceased individuals within the observation period.^2^

## Data availability statement

The datasets generated and analyzed during the current study are not publicly available due to existing data protection laws. Furthermore, data ownership is retained by the members of the UCDC and E‐IMD consortia making data available for specific research purposes. Data availability is subject to the consent of both consortia upon request.

## Statistical analysis

All analyses were performed using R (http://www.r-project.org). To evaluate the relationship between a continuous dependent variable and the cumulative residual enzymatic ASS1 activity as predictor variable, a linear regression or generalized additive regression model (GAM) with automated smoothing selections was used.^18^ In GAM, the linear relationship between the response variable and predictors are replaced by nonlinear smooth functions to evaluate an apparently non‐linear relationship between dependent and predictor variable. The R package “mgcv” was used to fit GAM regressions. To compare a numeric dependent variable between two groups, a *t*‐test with Welch correction was applied. We used unbiased recursive partitioning to determine cut‐off values for the impact of cumulative residual enzymatic ASS1 activity on outcome variables.^19^ Two groups were compared with a *t*‐test with Welch correction. *P* values reported were two‐sided. *P* ≤ 0.05 was considered statistically significant.

## Results

### Description of study population and ASS1 protein functions

We determined systematically enzymatic ASS1 activities in 71 individuals with CTLN1, representing 48 *ASS1* gene variants and 50 different combinations in total. Results of qRT‐PCR and Western Blot analyses of ASS1 mRNAs and proteins are depicted in Figures S1 and S2. Residual enzymatic ASS1 activities per variant combination are illustrated in Figure S3. Detailed descriptive characteristics of the study population for each subsequent analysis are shown in Tables S1 and S2. Of note, residual enzymatic ASS1 activity as determined in the biallelic expression system corresponded well with the enzymatic activity measured in patient fibroblasts and reported in the UCDC and E‐IMD databases (Table S3).

### Residual enzymatic ASS1 activity correlates with peak plasma ammonium and L‐citrulline concentrations during the first metabolic decompensation

**Figure 1:**
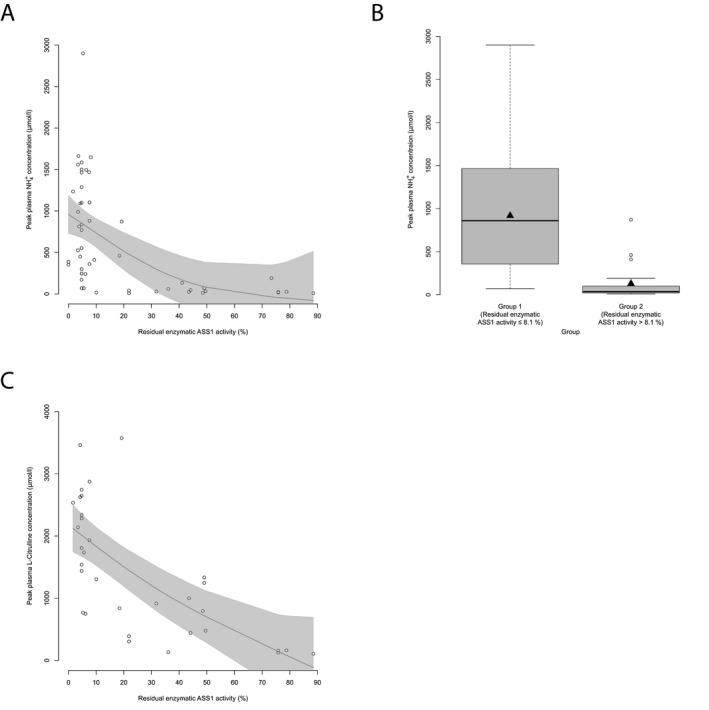
Residual enzymatic ASS1 activity correlates with initial biochemical parameters. (A) NH_4_^+^_max_ (*µ*mol/L) subject to residual enzymatic ASS1 activity (%) as determined in the biallelic expression system. Each point represents a single patient (*n* = 52). Gray line displays estimated regression curve. GAM‐analysis, *P* < 0.001, *R* ^2^ = 0.29. (B) Boxplot illustrating NH_4_^+^_max_ (*µ*mol/L) with a residual enzymatic ASS1 activity below or equal to 8.1% (*n* = 32) and above 8.1% (*n* = 20). Data are shown as median (black thick line) and mean (triangle), length of the box corresponds to interquartile range (IQR), upper and lower whiskers correspond to max. 1.5 × IQR, each point represents an outlier. Recursive partitioning, *P* < 0.001. (C) Peak plasma L‐citrulline concentration subject to residual enzymatic ASS1 activity (%). Each point represents a single patient (*n* = 33). Gray line displays estimated regression curve. GAM‐analysis, *P* < 0.001, *R* ^2^ = 0.46. ASS1, argininosuccinate synthetase 1.

Since decreased ASS1 activity results in hyperammonemia and hypercitrullinemia, we first studied whether NH_4_^+^_max_ and peak plasma L‐citrulline concentrations during the first metabolic decompensation are associated with residual enzymatic ASS1 activity. Notably, NH_4_^+^_max_ inversely correlated with the underlying residual enzymatic ASS1 activity (*n* = 52, *P* < 0.001, *R* ^2^ = 0.29, GAM‐analysis; Fig. 1A) as determined in the mammalian biallelic expression system. Furthermore, we identified a threshold distribution for individuals with CTLN1 with a residual enzymatic ASS1 activity below or equal to 8.1% exhibiting significantly higher NH_4_^+^_max_ at initial decompensation as opposed to patients with a residual enzymatic ASS1 activity above 8.1% (*n* = 52, *P* < 0.001, recursive partitioning; Fig. 1B). Even though patients with a residual enzymatic ASS1 activity below or equal to 8.1% have a higher probability of developing NH_4_^+^_max_ > 100 *µ*mol/L at initial decompensation, residual enzymatic activity does not allow to reliably predict the *exact* height of NH_4_^+^_max_ within this group (Fig. 1A). However, also peak plasma L‐citrulline concentration showed an inverse correlation with the underlying residual enzymatic ASS1 activity (*n* = 33, *P* < 0.001, *R* ^2^ = 0.46, GAM‐analysis; Fig. 1C).

### Residual enzymatic ASS1 activity correlates with the disease severity and cognitive outcome

**Figure 2:**
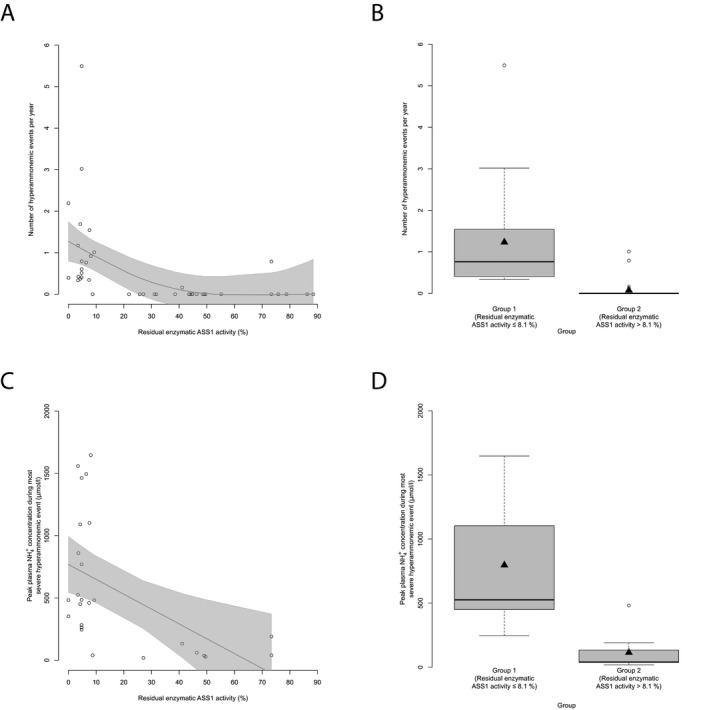
Residual enzymatic ASS1 activity predicts number and severity of HAEs. (A) Number of HAEs (NH_4_^+^_max_ ≥ 100 *µ*mol/L) per year subject to residual enzymatic ASS1 activity (%). Each point represents a single patient (*n* = 43). Gray line displays estimated regression curve. GAM‐analysis, *P* = 0.002, *R* ^2^ = 0.26. (B) Boxplot illustrating number of HAEs (NH_4_^+^_max_ ≥ 100 *µ*mol/L) per year with a residual enzymatic ASS1 activity below or equal to 8.1% (*n* = 17) or above 8.1% (*n* = 26). Data are shown as median (black thick line) and mean (triangle), length of the box corresponds to IQR, upper and lower whiskers correspond to max. 1.5 × IQR, each point represents an outlier. Recursive partitioning, *P* = 0.003. (C) NH_4_^+^_max_ during most severe HAE subject to residual enzymatic ASS1 activity (%). Each point represents a single patient (*n* = 26). Gray line displays estimated regression curve. GAM‐analysis, *P* = 0.007, *R* ^2^ = 0.23. (D) Boxplot illustrating NH_4_^+^_max_ during most severe HAE with a residual enzymatic ASS1 activity below or equal to 8.1% (*n* = 17) or above 8.1% (*n* = 9). Data are shown as median (black thick line) and mean (triangle), length of the box corresponds to IQR, upper and lower whiskers correspond to max. 1.5 × IQR, each point represents an outlier. Recursive partitioning, *P* = 0.01. ASS1, argininosuccinate synthetase 1.

**Figure 3:**
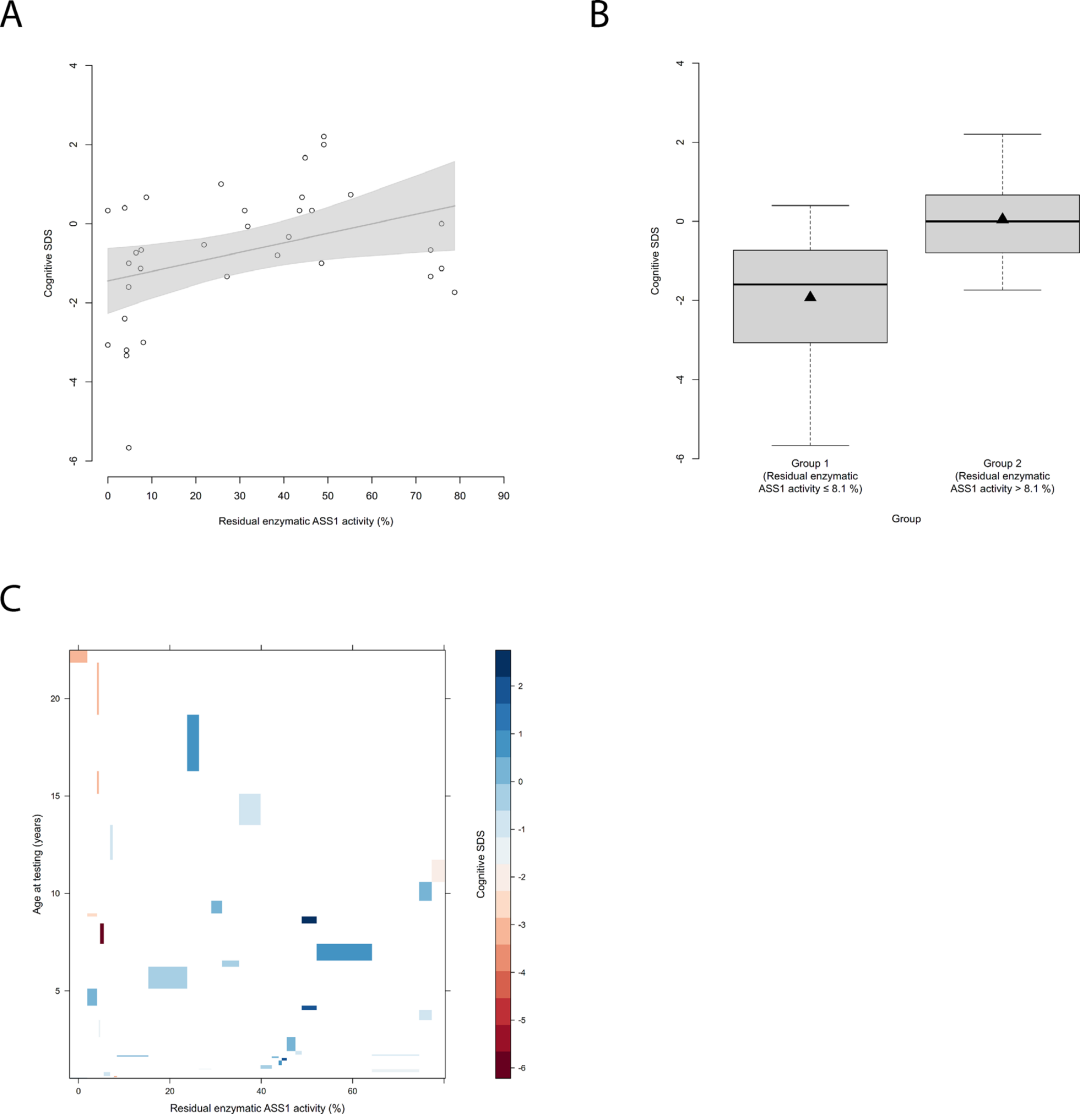
Residual enzymatic ASS1 activity correlates with cognitive outcome. (A) Cognitive SDS subject to residual enzymatic ASS1 activity (%). Each point represents a single patient (*n* = 34). Grey line indicates linear regression curve. Linear regression, *P* = 0.029, *R* ^2^ = 0.14. (B) Boxplot illustrating cognitive SDS with a residual enzymatic ASS1 activity below or equal to 8.1% (*n* = 13) and above 8.1% (*n* = 21). Data are shown as median (black thick line) and mean (triangle), length of the box corresponds to IQR, upper and lower whiskers correspond to max. 1.5 × IQR. Recursive partitioning, *P* = 0.031. (C) Levelplot for cognitive SDS, residual enzymatic ASS1 activity and age at testing (years). Cognitive SDS values are indicated by color coding in grading from blue to red with descending cognitive SDS. ASS1, argininosuccinate synthetase 1.

Subsequently, we assessed whether residual enzymatic ASS1 activity correlates with the clinical disease course as indicated by reported number of HAEs per year, NH_4_^+^_max_ during the most severe hyperammonemic decompensation and the cognitive SDS of individuals with CTLN1 at their most recent visit. Individuals with a residual enzymatic ASS1 activity below or equal to 8.1% not only had a significantly higher number of HAEs per year (*n* = 43, *P* = 0.003, recursive partitioning) and higher NH_4_^+^_max_ during the most severe hyperammonemic decompensation (*n* = 26, *P* = 0.01, recursive partitioning) (Fig. 2A–D), but also exhibited lower cognitive function as opposed to individuals above this threshold (*n* = 34, *P* = 0.031, recursive partitioning) (Fig. 3A and B). Intriguingly, besides residual enzyme activity also age seems to affect cognitive functions since older individuals with CTLN1 showed lower cognitive SDS than younger individuals (Fig. 3C).

### Residual enzymatic ASS1 activity is associated with clinical outcome parameters

**Figure 4:**
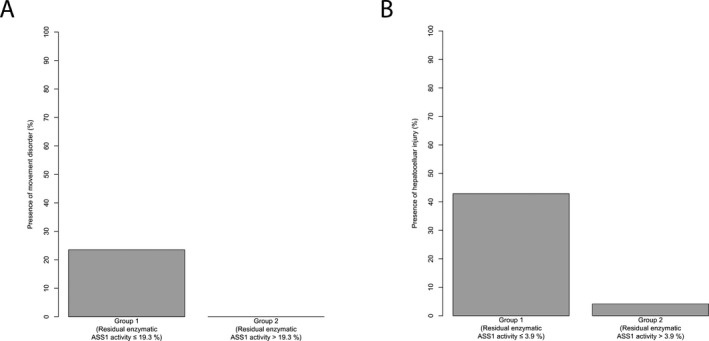
Residual enzymatic ASS1 activity predicts organ‐specific manifestations. (A) Boxplot illustrating presence of movement disorders (%) for individuals with a residual enzymatic ASS1 activity below or equal to 19.3% (*n* = 34) and above 19.3% (*n* = 26). Gray shading corresponds to individuals with movement disorders in each group. Recursive partitioning, *P* = 0.03. (B) Boxplot displaying presence of hepatocellular injury (%) for individuals with a residual enzymatic ASS1 activity below or equal to 3.9% (*n* = 7) and above 3.9% (*n* = 48). Gray shading corresponds to individuals with episode(s) of hepatocellular injury in each group. Recursive partitioning, *P* = 0.039. ASS1, argininosuccinate synthetase 1.

Given the above described correlations of residual enzymatic ASS1 activity with the metabolic disease course and cognitive outcome, we next investigated, whether this observation also holds true for other clinical features like movement disorder, tone change, hepatocellular injury, and kidney dysfunction. Indeed, residual enzymatic ASS1 activity is associated with the presence of movement disorder and hepatocellular injury. Affected individuals with a residual enzymatic ASS1 activity below or equal to 19.3% suffer more often from movement disorders (*n* = 60, *P* = 0.03, recursive partitioning; Fig. 4A). This observation could be corroborated for the presence of hepatocellular injury with a residual enzymatic ASS1 activity of 3.9% as threshold (*n* = 55, *P* = 0.039, recursive partitioning; Fig. 4B). However, residual enzymatic ASS1 activity did not discriminate between individuals with or without tone changes (*n* = 65, *P* = 0.16, *t*‐test) neither between CTLN1‐individuals with (FAS‐GFR < 90 mL/min/1.73 m^2^) or without (FAS‐GFR ≥ 90 mL/min/1.73 m^2^) reported episodes of impaired kidney function (*n* = 47, *P* = 0.44, *t*‐test).

### Empiric clinical practice reflects disease severity

**Figure 5:**
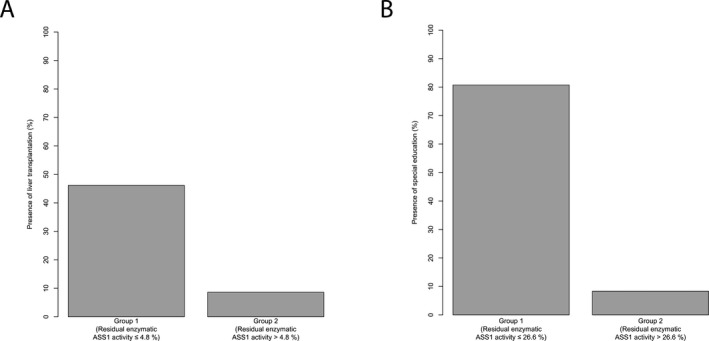
Decision for liver transplantation and special education reflects risk‐stratification by residual enzymatic ASS1 activity. (A) Boxplot illustrating proportion of individuals (%) with a liver graft for those with a residual enzymatic ASS1 activity below or equal to 4.8% (*n* = 13) and above 4.8% (*n* = 58). Grey shading corresponds to individuals with liver graft in each group. Recursive partitioning, *P* = 0.011. (B) Boxplot displaying proportion of individuals (%) with special education for those with a residual enzymatic ASS1 activity below or equal to 26.6% (*n* = 26) and above 26.6% (*n* = 12). Gray shading corresponds to individuals with special education in each group. Recursive partitioning, *P* < 0.001. ASS1, argininosuccinate synthetase 1.

Since residual enzymatic ASS1 activity predicts the disease course and clinical outcome in CTLN1, we tested whether the current clinical practice as indicated by LTx status and implementation of special education in the management might also correlate with residual enzymatic ASS1 activity. In fact, individuals with a residual enzymatic ASS1 activity below or equal to 4.8% underwent LTx more often than those with higher activity (*n* = 71, *P* = 0.011, recursive partitioning; Fig. 5A). Noteworthy, individuals with a residual enzymatic ASS1 activity above 10% did not undergo LTx during the observation period at all. Furthermore, individuals with a residual enzymatic ASS1 activity below or equal to 26.6% received special education support more frequently (*n* = 38, *P* < 0.001, recursive partitioning; Fig. 5B) than above this threshold.

## Discussion

### A novel mammalian biallelic expression system predicts the functional consequences of pathogenic *ASS1* variants

Using a newly established mammalian biallelic expression system for the quantitative analysis of residual enzymatic ASS1 activity based on pathogenic *ASS1* variants reported in the UCDC and E‐IMD registries, we show, that residual enzymatic ASS1 activity reliably predicts the clinical disease course and cognitive outcome in CTLN1. Individuals with a residual enzymatic ASS1 activity below or equal to 8% experience a higher number and more severe HAEs, and have a lower cognitive outcome at most recent visit.

The established mammalian biallelic expression system combines the overexpression of patient‐specific variants in ASS1 proteins in COS‐7 cells and the quantification of residual enzymatic ASS1 activities in cell lysates 48 h after transfection applying a novel coupled ASS1 enzyme assay. Intriguingly, the residual enzymatic ASS1 activities as determined by our assay show a correlation with biochemical and clinical endpoints, thereby confirming that the modelling approach quantitatively reflects the protein dysfunction in individuals with CTLN1. This observation might owe to the overexpression strategy of equimolar transfection of both (homozygous/compound heterozygous) ASS1 expression vectors, which as a consequence most likely leads to an equimolar expression of the (mutated) proteins (encoded by both alleles), mimicking the endogenous situation in individuals with CTLN1. The utilized COS‐7 cell line has been used as overexpression system for the investigation of protein (dys‐) functions for more than three decades including the quantification of residual activities of enzymes of intermediary metabolism.^20^ Coupled enzyme assays have been successfully established and are widely used for the reliable determination of activities of glycolytic or TCA cycle enzymes as well as respiratory chain complexes, which allow the real‐time quantification of activity rates by exploiting redox‐reactions of redox‐active coenzymes such as NAD or FAD.^21^, ^22^, ^23^ This strategy also proved effective for the quantitative analysis of residual enzymatic ASS1 activity in our study. The established mammalian biallelic assay system is a non‐invasive modelling approach, which enables the systematic investigation of the cumulative effect of reported homozygous and most importantly compound heterozygous mutations within the *ASS1* gene in a standardized and time‐efficient manner on a highly discriminative scale, in particular for low residual enzymatic ASS1 activities.

### From bench to bedside – modelling evidence‐based thresholds for clinical endpoints

The subsequent correlation analysis between residual enzymatic ASS1 activity and biochemical, clinical as well as organ‐specific endpoints is prototypic for UCDs and a powerful tool for individuals with CTLN1 since (1) it identifies robust cut‐offs of residual enzymatic ASS1 activity for the prediction of clinical outcomes, (2) it might serve as a tool for physicians in counselling of patients and their families, (3) it is a prerequisite for the development of risk‐stratified, individualized concepts for therapy and care, (4) it is likely to be causally related to the disease course and neurological outcome, and (5) it reduces bias and misinterpretation of future studies aiming at evaluating the impact of newborn screening programs and innovative therapies on the health outcomes of affected individuals.

Interestingly, residual enzymatic ASS1 activity of 8% or less is associated with a higher NH_4_^+^_max_ at initial decompensation, and a higher number and severity of HAEs. This is of importance, since these parameters have recently been reported as major risk factors for a poor outcome.^5^, ^7^, ^8^, ^24^, ^25^ Intriguingly, individuals with CTLN1 and a residual enzymatic ASS1 activity of 8% or less develop approximately 1.25 HAEs per year despite adequate conventional metabolic therapy, whereas this is not the case for those with higher residual enzymatic ASS1 activity suffering from HAEs only once every 12.5 years in mean. However, there are single individuals who do also show high NH_4_^+^_max_ at initial decompensation with a maximum of approx. 880 *µ*mol/L (Fig. 1B and Table S1) and do decompensate once per year at maximum despite residual enzymatic ASS1 activity higher than 8% (Fig. 2B and Table S1), which needs to be taken into account by physicians for the clinical management of these patients. Importantly, the threshold of 8% is also reflected by the susceptibility to suffer from an impaired cognitive outcome.

Given known variables of an unfavorable clinical outcome (such as initial NH_4_^+^_max_, number and severity of HAEs) and an evidence‐based cut‐off value of 8% or below predicting a poor cognitive outcome, we propose residual enzymatic ASS1 activity of 8% or below, as assessed by our system, as a reasonable threshold for discriminating individuals with a severe (≤ 8%) or mild‐to‐moderate (> 8%) clinical phenotype. This is corroborated by the finding that in our cohort only one CTLN1‐individual above 8% residual enzymatic ASS1 activity underwent LTx. Of note, the reported thresholds as determined by our standardized assay might slightly differ for the investigated mutations when compared to other methods.

Even though residual enzymatic ASS1 activity correlates well with the overall clinical disease course and cognitive outcome of CTLN1‐individuals, the positive predictive value does not reach 100% for any analyzed clinical variable. For the cognitive outcome, 46% of patients with a residual enzymatic ASS1 activity below the threshold of 8% have a cognitive SDS below or equal to −2, whereas 54% of patients perform better in cognitive testing. The observation, that clinical manifestations are not present in all individuals below the defined thresholds of residual enzymatic ASS1 activity can also be corroborated for further outcome variables, such as movement disorder, and hepatocellular injury. These findings indicate alternative modifying disease mechanisms apart from residual enzymatic ASS1 activity underlying the clinical course of CTLN1‐individuals. Notably, the enzyme ASS1 has recently been identified as potential RNA‐binding protein in RNA interactome capture.^26^ These newly identified RNA‐binding properties of ASS1 might be implicated in the pathophysiology of CTLN1, which needs to be addressed in future research. Moreover, the observation that residual enzymatic ASS1 activity does not allow to predict the *exact* NH_4_^+^_max_ at initial decompensation as well as the *exact* number of HAEs within the group of patients below the threshold of 8% indicates that – besides residual enzymatic activity – further precipitating factors (e.g., severity of catabolic state, delay between first symptoms and treatment initiation, compliance to long‐term treatment) might determine the severity of metabolic decompensations, as reflected by the scattered data in this group. Another possible explanation might be, that a subset of patients have “leaky mutations” associated with altered kinetic properties (Km mutations) due to decreased substrate binding of the ASS1 enzyme,^27^ which needs to be addressed in future research.

### Residual enzymatic ASS1 activity qualifies as predictive biomarker for individuals with CTLN1

Biomarkers are defined to reflect the activity of a disease process and can be of diagnostic, prognostic, predictive, or pharmacodynamic nature.^28^, ^29^ Specifically, residual enzymatic ASS1 activity as determined in our mammalian biallelic expression system can be considered a prognostic and predictive biomarker based on the present findings. In contrast, biomarkers serving as truly validated surrogate markers are required to capture the severity of disease, which is the case for residual enzymatic ASS1 activity, but in addition need to reflect the net effect of treatment on the true outcome.^30^ Since there are currently no specific therapies available, which aim at increasing the residual enzymatic activity of the ASS1 enzyme in CTLN1, the latter requirement of demonstrating the reflection of net treatment effects is not possible at the current time. Nevertheless, the identification that the clinical disease course and cognitive outcome clearly correlates with the residual enzymatic ASS1 activity is encouraging and might stimulate future drug development. Increasing the residual enzymatic ASS1 activity above a threshold of 8% (e.g., by application of chaperones or antisense nucleotides targeting aberrant splicing of the precursor mRNA to increase the level of normally translated protein) as demonstrated for other inherited diseases might have a major beneficial effect on the disease course, by reducing the number and severity of HAEs, and by improving the cognitive and hepatic function of affected individuals with CTLN1 as implicated by our data.^31^, ^32^

Preliminary data suggest that height of the initial peak plasma L‐citrulline concentration inversely correlates with residual enzymatic ASS1 activity (Fig. S4), which has to be proven in future prospective trials, in particular with regard to L‐citrulline concentrations determined in dried blood spots during newborn screening. Furthermore, our functional data propose a new severity‐adjusted classification system for individuals with CTLN1, which has to be regarded complementary to the current classification based on the onset type (Fig. S5). Importantly, clinicians should be aware that patients who are defined asymptomatic to date might develop symptoms over time, which has already been described for urea cycle disorders.^2^ Further intra‐individual long‐term data will enable to determine to which extent this also applies for the investigated study cohort.

### Limitations

This study has some inherent limitations. Only exonic mutations have been modelled and investigated, due to the applied technique of cloning the coding sequence of the *ASS1* gene into the mammalian expression vector, which does not allow the investigation of intronic mutations associated with defective splicing. Our analysis does not claim to systematically investigate the underlying pathomechanisms of specific mutations leading to decreased residual enzymatic ASS1 activity on an individual level (e.g., impaired protein folding, decreased protein stability, etc.). Figures S1 and S2 depicting results of qRT‐PCR data and protein expression levels as determined by Western blot analysis should be considered exploratory and might serve as a basis for future detailed investigations regarding underlying mechanisms of reduced residual enzymatic ASS1 activity for each mutation. Moreover, in light of potential disease progression in CTLN1 over time further intra‐individual long‐term data is necessary to substantiate our findings, in particular with regard to progressive cognitive deterioration in older (or asymptomatic) individuals with CTLN1.

## Conclusion

Based on the biallelic expression system, we propose a new severity‐adjusted classification system for individuals with CTLN1 having a severe (residual enzymatic ASS1 activity ≤ 8%) and mild‐to‐moderate (residual enzymatic ASS1 activity > 8%) phenotype, which is to be regarded complementary to the currently existing clinical classification system for CTLN1. Future analyses will prove, if an intermediate phenotype might be identified in the group of CTLN1‐individuals with residual enzymatic ASS1 activity above 8%. Given the evident impact of the present method for the prediction of the clinical disease course and cognitive outcome, we are hopeful that this modelling approach can be applied to other inborn errors of metabolism.

## Author Contributions

The STROBE statement was used when preparing this manuscript. MZ and RP contributed to conception and design of the study. MZ, SK, FG, NS, SCSN, ALG, GFH, SFG, RP, and all individual contributors from the UCDC and E‐IMD consortia study group (Table S4) contributed to acquisition and analysis of data. MZ, SK, SCSN, SFG, and RP contributed to drafting the text and preparing the figures.

## Conflict of Interest

SK receives funding from Horizon Pharma Ireland Limited for the European Post‐Authorization Registry for Ravicti® (glycerol phenylbutyrate) oral liquid in partnership with the E‐IMD (RRPE) (EU PAS Register no. EUPAS17267; http://www.encepp.eu/). The sponsors have in no way influenced the design, conductance, analysis and report of the present study. All other authors declare that they have no conflict of interest.

## Supporting information


**Figure S1** **.** Overview of relative mRNA expression levels per variant combination.
**Figure S2**. Overview of protein expression levels per variant combination.
**Figure S3** **.** Overview of residual enzymatic ASS1 activities per variant combination.
**Figure S4** **.** Peak plasma L‐citrulline concentration (at initial decompensation) reflects residual enzymatic ASS1 activity (%).
**Figure S5** **.** Correlation between disease onset (EO, LO, Asymptomatic) and residual enzymatic ASS1 activity (%).
**Table S1** **.** Descriptive characteristics for correlation analyses in CTLN 1 – part I.
**Table S2** **.** Descriptive characteristics for correlation analyses in CTLN1 – part II.
**Table S3** **.** Comparison of residual enzymatic ASS1 activity determined in the biallelic expression system and patient fibroblasts.
**Table S4** **.** Additional members and affiliations of the UCDC and E‐IMD consortia study group.
