# Supplementary material for: Early prediction of phenotypic severity in Citrullinemia Type 1
Source: Ann Clin Transl Neurol. 2019 Aug 30;6(9):1858–71. doi: 10.1002/acn3.50886 (PMC6764635; doi:10.1002/acn3.50886)
Supplement: Supplementary file 1 — Figure S1 . Overview of relative mRNA expression levels per variant combination. Figure S2. Overview of protein expression levels per variant combination. Figure S3 . Overview of residual enzymatic ASS1 activities per variant combination. Figure S4 . Peak plasma L‐citrulline concentration (at initial decompensation) reflects residual enzymatic ASS1 activity (%). Figure S5 . Correlation between disease onset (EO, LO, Asymptomatic) and residual enzymatic ASS1 activity (%). Table S1 . Descriptive characteristics for correlation analyses in CTLN 1 – part I. Table S2 . Descriptive characteristics for correlation analyses in CTLN1 – part II. Table S3 . Comparison of residual enzymatic ASS1 activity determined in the biallelic expression system and patient fibroblasts. Table S4 . Additional members and affiliations of the UCDC and E‐IMD consortia study group. [file ACN3-6-1858-s001.pdf]

Supplementary Figure 1

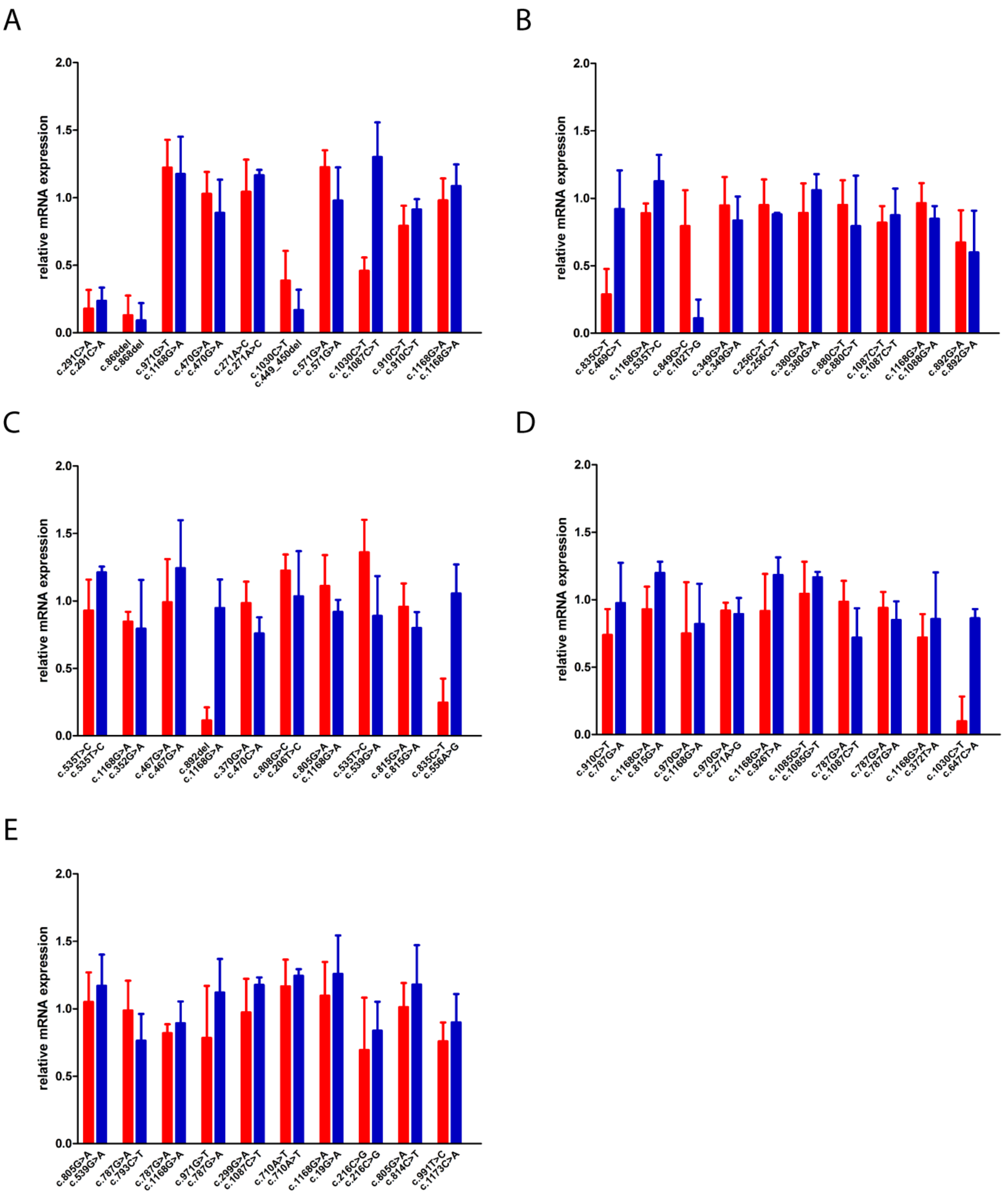

Supplementary Figure 2

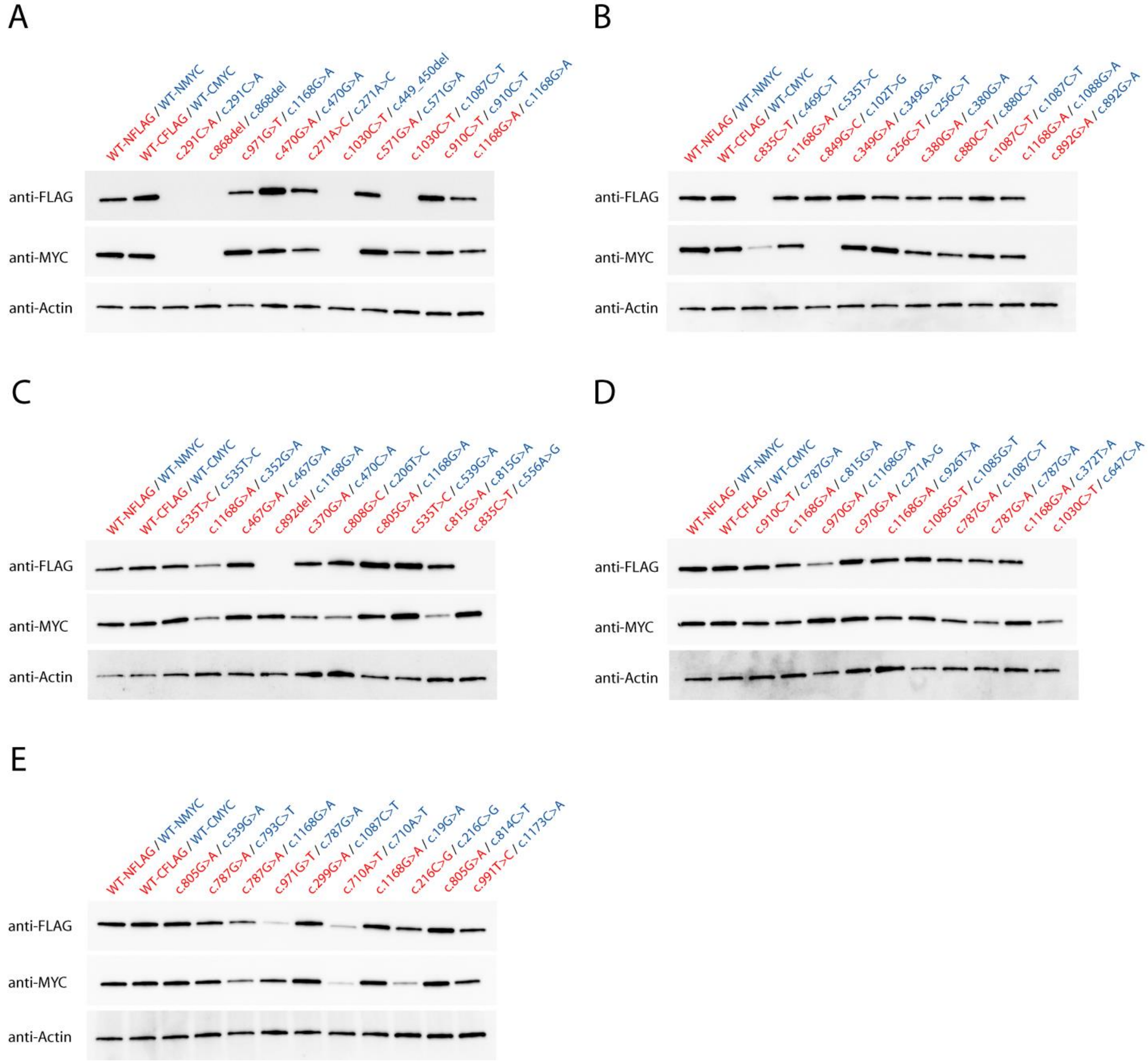

Supplementary Figure 3

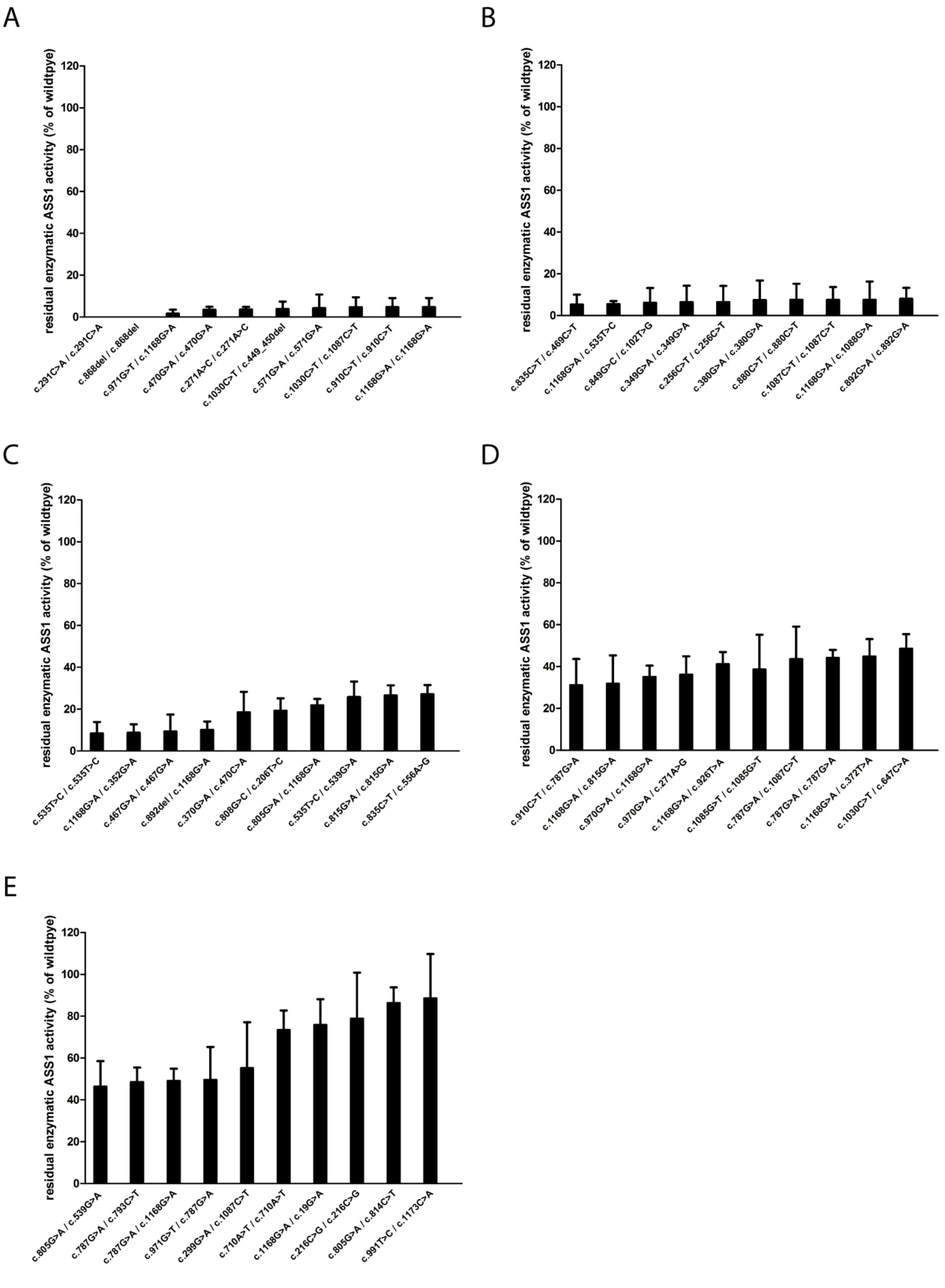

Supplementary Figure 4

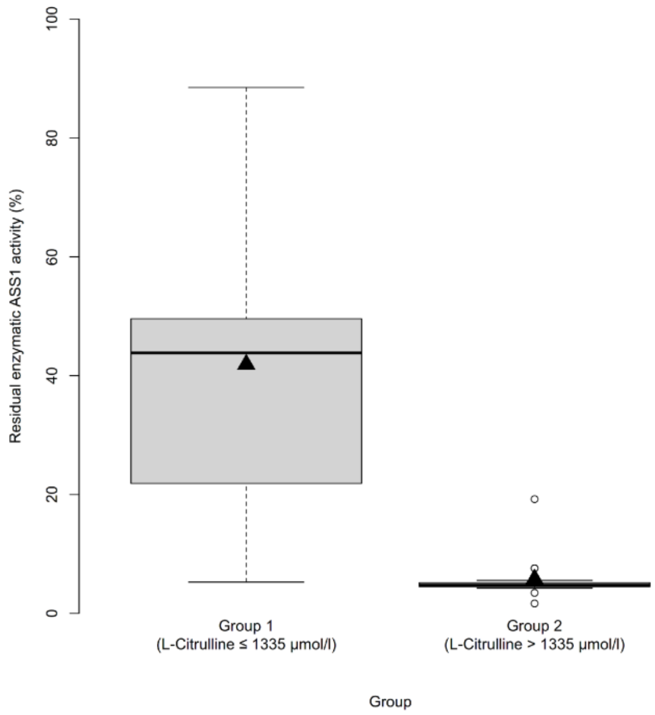

Supplementary Figure 5

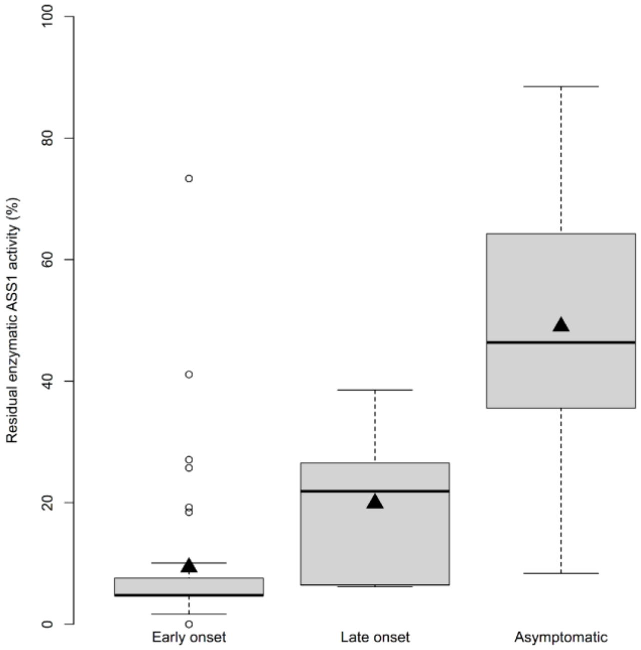

Supplementary Table 1. Descriptive characteristics for correlation analyses in CTLN 1 – part I

**Correlation between residual ASS1 activity and peak plasma NH_4^+ concentration**			
**ASS1 activity (%)**<br><br>*Mean, SD*<br>*Median [Q1, Q3]*<br>*Min, Max; n*	**Peak plasma NH_4^+ concentration (µmol/l)**<br><br>*Mean, SD*<br>*Median [Q1, Q3]*<br>*Min, Max; n*	**Peak plasma NH_4^+ concentration (µmol/l), if ASS1 activity ≤ 8.1%**<br><br>*Mean, SD*<br>*Median [Q1, Q3]*<br>*Min, Max; n*	**Peak plasma NH_4^+ concentration (µmol/l), if ASS1 activity > 8.1%**<br><br>*Mean, SD*<br>*Median [Q1, Q3]*<br>*Min, Max; n*
20.0, 24.4<br>6.3 [4.8, 32.9]<br>0, 88.5; 52	613.7, 645.5<br>373.9 [66.7, 1093.5]<br>9, 2900; 52	917.4, 639.7<br>859.5 [358.8, 1464.7]<br>69.3, 2900; 32	127.8, 216.5<br>37.0 [21.5, 85.9]<br>9.0, 872.0; 20
**Correlation between residual ASS1 activity and peak plasma L-citrulline concentration**			
**ASS1 activity (%)**<br><br>*Mean, SD*<br>*Median [Q1, Q3]*<br>*Min, Max; n*	**Peak plasma L-citrulline concentration (µmol/l)**<br><br>*Mean, SD*<br>*Median [Q1, Q3]*<br>*Min, Max; n*	**Peak plasma L-citrulline concentration (µmol/l), if ASS1 activity ≤ 19.3%**<br><br>*Mean, SD*<br>*Median [Q1, Q3]*<br>*Min, Max; n*	**Peak plasma L-citrulline concentration (µmol/l), if ASS1 activity > 19.3%**<br><br>*Mean, SD*<br>*Median [Q1, Q3]*<br>*Min, Max; n*
25.5, 26.3<br>10.1 [4.8, 44.1]<br>1.7, 88.5; 33	1422.4, 1030.2<br>1305.0 [478.0, 2283.0]<br>108.0, 3574.0; 33	2078.6, 839.7<br>2140.0 [1490.0, 2636.5]<br>751.0, 3574.0; 19	542.6, 434.3<br>416.5 [160.0, 886.0]<br>108.0, 1335.0; 14
**Correlation between residual ASS1 activity and number of hyperammonemic events (HAE) per year**			
**ASS1 activity (%)**<br><br>*Mean, SD*<br>*Median [Q1, Q3]*<br>*Min, Max; n*	**Number of HAE per year**<br><br>*Mean, SD*<br>*Median [Q1, Q3]*<br>*Min, Max; n*	**Number of HAE per year, if ASS1 activity ≤ 8.1%**<br><br>*Mean, SD*<br>*Median [Q1, Q3]*<br>*Min, Max; n*	**Number of HAE per year, if ASS1 activity > 8.1%**<br><br>*Mean, SD*<br>*Median [Q1, Q3]*<br>*Min, Max; n*
31.1, 27.7<br>27.1 [4.8, 48.8]<br>0, 88.5; 43	0.53, 1.02<br>0 [0, 0.68]<br>0, 5.5; 43	1.23, 1.33<br>0.76 [0.42, 1.54]<br>0.34, 5.5; 17	0.08, 0.25<br>0 [0, 0]<br>0, 1.0; 26
**Correlation between residual ASS1 activity and peak plasma NH_4^+ concentration during severest HAE**			
**ASS1 activity (%)**<br><br>*Mean, SD*<br>*Median [Q1, Q3]*<br>*Min, Max; n*	**Peak plasma NH_4^+ concentration (µmol/l)**<br><br>*Mean, SD*<br>*Median [Q1, Q3]*<br>*Min, Max; n*	**Peak plasma NH_4^+ concentration (µmol/l), if ASS1 activity ≤ 8.1%**<br><br>*Mean, SD*<br>*Median [Q1, Q3]*<br>*Min, Max; n*	**Peak plasma NH_4^+ concentration (µmol/l), if ASS1 activity > 8.1%**<br><br>*Mean, SD*<br>*Median [Q1, Q3]*<br>*Min, Max; n*
17.5, 22.7<br>5.6 [4.4, 22.7]<br>0, 73.3; 26	560.4, 524.8<br>455.0 [148.3, 837.5]<br>19.0, 1647.0; 26	796.4, 498.3<br>526.0 [450.0, 1103.0]<br>245.44, 1647.0; 17	114.7, 149.1<br>40.0 [37.0, 134.0]<br>19.0, 482.0; 9
**Correlation between residual ASS1 activity and cognitive SDS at last regular visit**			
**ASS1 activity (%)**<br><br>*Mean, SD*<br>*Median [Q1, Q3]*<br>*Min, Max; n*	**Cognitive SDS**<br><br>*Mean, SD*<br>*Median [Q1, Q3]*<br>*Min, Max; n*	**Cognitive SDS, if ASS1 activity ≤ 8.1%**<br><br>*Mean, SD*<br>*Median [Q1, Q3]*<br>*Min, Max; n*	**Cognitive SDS, if ASS1 activity > 8.1%**<br><br>*Mean, SD*<br>*Median [Q1, Q3]*<br>*Min, Max; n*
30.7, 25.9<br>29.1 [5.2, 48.0]<br>0, 78.8; 34	-0.7, 1.7<br>-0.7 [-1.3, 0.3]<br>-5.7, 2.2; 34	-1.9, 1.7<br>-1.6 [-3.1, -0.7]<br>-5.7, 0.4; 13	0.1, 1.1<br>0 [-0.8, 0.7]<br>-1.7, 2.2; 21

Supplementary Table 2. Descriptive characteristics for correlation analyses in CTLN1 – part II

Correlation between residual ASS1 activity and occurrence of movement disorder (MD)			
**ASS1 activity of individuals with MD (%)**<br>*Mean, SD*<br>*Median [Q1, Q3]*<br>*Min, Max; n*	**ASS1 activity of individuals w/o MD (%)**<br>*Mean, SD*<br>*Median [Q1, Q3]*<br>*Min, Max; n*	**MD, if ASS1 activity ≤ 19.3% (% of total)**	**MD, if ASS1 activity > 19.3% (% of total)**
6.7, 5.6<br>4.8 [4.8, 7.7]<br>0, 19.2; 8	26.4, 24.5<br>20.2 [5.5, 44.3]<br>1.7, 88.5; 52	8/34 = 23.5%	0/26 = 0%
**Correlation between residual ASS1 activity and occurrence of hepatocellular injury (HCI)**			
**ASS1 activity of individuals with HCI (%)**<br>*Mean, SD*<br>*Median [Q1, Q3]*<br>*Min, Max; n*	**ASS1 activity of individuals w/o HCI (%)**<br>*Mean, SD*<br>*Median [Q1, Q3]*<br>*Min, Max; n*	**HCI, if ASS1 activity ≤ 3.9% (% of total)**	**HCI, if ASS1 activity > 3.9% (% of total)**
3.2, 3.3<br>3.84 [0, 4.8]<br>0, 7.6; 5	28.9, 26.7<br>21.9 [4.9, 46.0]<br>1.7, 88.5; 50	3/7 = 42.9%	2/48 = 4.2%
**Correlation between residual ASS1 activity and liver transplantation (LTx) status**			
**ASS1 activity of individuals with LTx (%)**<br>*Mean, SD*<br>*Median [Q1, Q3]*<br>*Min, Max; n*	**ASS1 activity of individuals w/o LTx (%)**<br>*Mean, SD*<br>*Median [Q1, Q3]*<br>*Min, Max; n*	**LTx, if ASS1 activity ≤ 4.8% (% of total)**	**LTx, if ASS1 activity > 4.8% (% of total)**
5.4, 2.1<br>4.7 [3.5, 7.0]<br>3.5, 9.3; 11	26.0, 25.5<br>14.3 [4.8, 44.3]<br>0, 88.5; 60	6/13 = 46.2%	5/58 = 8.6%
**Correlation between residual ASS1 activity and special education**			
**ASS1 activity of individuals with special education (%)**<br>*Mean, SD*<br>*Median [Q1, Q3]*<br>*Min, Max; n*	**ASS1 activity of individuals w/o special education (%)**<br>*Mean, SD*<br>*Median [Q1, Q3]*<br>*Min, Max; n*	**Special education, if ASS1 activity ≤ 26.6% (% of total)**	**Special education, if ASS1 activity > 26.6% (% of total)**
10.4, 16.3<br>4.8 [4.7, 7.9]<br>0, 78.8; 22	35.1, 22.8<br>35.2 [21.0, 49.1]<br>3.9, 75.8; 16	21/26 = 80.8%	1/12 = 8.3%

Supplementary Table 3.

Genotype	Residual enzymatic ASS1 activity (expression system) [% of wildtype]	Residual enzymatic ASS1 activity (fibroblasts) [% of normal]
c.470G>A / c.470G>A	3.4	3
c.1168G>A / c.1168G>A	4.8	1
c.256C>T / c.256C>T	6.5	5
c.535T>C / c.535C>T	8.4	7
c.271A>G / c.970G>A	36.1	25

Supplementary Table 4.

UCDC consortium (to be listed in PubMed in alphabetical order)
Marc Yudkoff, University of Pennsylvania School of Medicine and Children’s Hospital of Philadelphia, Philadelphia, Pennsylvania, USA
Irma Payan-Walters, The Children’s Hospital of Philadelphia, Division of Human Genetics and Metabolic Disease Program, Philadelphia, Pennsylvania, USA
Nicholas Ah Mew, Children's National Health System, Washington, DC, USA
Andreas Schulze, The Hospital for Sick Children and University of Toronto, Toronto, Ontario, Canada
Jennifer Seminara, Children’s National Health System, Washington, District of Columbia, USA
Lindsay C. Burrage, Department of Molecular and Human Genetics, Baylor College of Medicine and Texas Children’s Hospital, Houston, Texas, USA
J. Lawrence Merritt II, University of Washington and Seattle Children’s Hospital, Seattle, Washington, USA
Matthias R. Baumgartner, University Children’s Hospital Zurich and Children’s Research Center, Zurich, Switzerland
Jirair K. Bedoyan, Center for Human Genetics and Department of Genetics and Genome Sciences, University Hospitals Cleveland Medical Center and Case Western Reserve University, Cleveland, Ohio, USA
Susan A. Berry, University of Minnesota, Minneapolis, Minnesota, USA
Suzanne Hollander, David Geffen School of Medicine at UCLA, Los Angeles, California, USA
Gerard T. Berry, Harvard Medical School and Boston Children’s Hospital, Boston, Massachusetts, USA
George A. Diaz, Mount Sinai School of Medicine, Department of Genetics and Genomics Sciences, New York, NY, USA

E-IMD registry (to be listed in PubMed in alphabetical order)
Javier Blasco-Alonso, Hospital Materno-Infantil, AVda Arroyo de los Ángeles s/n; 29011 , Málaga, Spain
Angeles Garcia-Cazorla, Hospital San Joan de Deu, Institut Pediàtric de Recerca. Servicio de Neurologia and CIBERER, ISCIII, Barcelona, Spain
Peter Freisinger, Klinik für Kinder- und Jugendmedizin, Klinikum am Steinenberg, Reutlingen, Germany
Elisa Leão Teles, Unidade de Doenças Metabólicas, Serviço de Pediatria, Centro Hospitalar Universitário de S. João EPE, Porto, Portugal
Peter M. van Hasselt, Wilhelmina Children's Hospital, Utrecht, The Netherlands
Anastasia Skouma, Institute of Child Health, Athens, Greece
Allan Melgaard Lund, Centre Inherited Metabolic Diseases, Departments of Paediatrics and Clinical Genetics, Copenhagen University Hospital, Rigshospitalet, Copenhagen, Denmark
Anil Jalan, NIRMAN, Biochemical Genetics, Om Rachana Society, Mumbai, India
Roshni Vara, Department of Inherited Metabolic Disease, Evelina Children's Hospital, St Thomas' Hospital, London, United Kingdom
Corinne De Laet, Hôpital Universitaire des Enfants Reine Fabiola, Université Libre de Bruxelles, Brussels, Belgium
Manuel Schiff, Robert-Debré University Hospital, Reference Center for Inborn Errors of Metabolism, Paris, France
Carlo Dionisi-Vici, Ospedale Pediatrico Bambino Gesù, U.O.C. Patologia Metabolica, Rome, Italy
Adrijan Sarajlija, Mother and Child Health Care Institute of Serbia, Department of Metabolism and Clinical Genetics and University of Belgrade, School of Medicine, Belgrade, Serbia
Andrew Morris, Manchester University Hospitals NHS Foundation Trust & Manchester Academic Health Sciences Centre, Willink Biochemical Genetics Centre, St Mary's Hospital, Manchester, United Kingdom
Nastassja Himmelreich, Center for Pediatric and Adolescent Medicine, Division of Pediatric Neurology and Metabolic Medicine, University Hospital Heidelberg, Im Neuenheimer Feld 430, 69120 Heidelberg, Germany
